# The Impact of Environmental Factors on the Secretion of Gastrointestinal Hormones

**DOI:** 10.3390/nu17152544

**Published:** 2025-08-02

**Authors:** Joanna Smarkusz-Zarzecka, Lucyna Ostrowska, Marcelina Radziszewska

**Affiliations:** Department of Dietetics and Clinical Nutrition, Medical University of Bialystok, 15-054 Bialystok, Poland; joanna.smarkusz-zarzecka@umb.edu.pl (J.S.-Z.);

**Keywords:** environmental factors, gastrointestinal hormones, obesity, somatostatin, gastrin, ghrelin, secretin, GLP-1, GIP, PYY, CCK

## Abstract

The enteroendocrine system of the gastrointestinal (GI) tract is the largest endocrine organ in the human body, playing a central role in the regulation of hunger, satiety, digestion, and energy homeostasis. Numerous factors—including dietary components, physical activity, and the gut microbiota—affect the secretion of GI hormones. This study aims to analyze how these factors modulate enteroendocrine function and influence systemic metabolic regulation. This review synthesizes the current scientific literature on the physiology and distribution of enteroendocrine cells and mechanisms of hormone secretion in response to macronutrients, physical activity, and microbial metabolites. Special attention is given to the interactions between gut-derived signals and central nervous system pathways involved in appetite control. Different GI hormones are secreted in specific regions of the digestive tract in response to meal composition and timing. Macronutrients, particularly during absorption, stimulate hormone release, while physical activity influences hormone concentrations, decreasing ghrelin and increasing GLP-1, PYY, and leptin levels. The gut microbiota, through fermentation and metabolite production (e.g., SCFAs and bile acids), modulates enteroendocrine activity. Species such as *Akkermansia muciniphila* are associated with improved gut barrier integrity and enhanced GLP-1 secretion. These combined effects contribute to appetite regulation and energy balance. Diet composition, physical activity, and gut microbiota are key modulators of gastrointestinal hormone secretion. Their interplay significantly affects appetite regulation and metabolic health. A better understanding of these relationships may support the development of personalized strategies for managing obesity and related disorders.

## 1. Introduction

In the human body, there are numerous types of enteroendocrine cells that differ in the types of hormones they secrete. These cells are located within the epithelium of the gastrointestinal (GI) tract along its entire length, from the stomach to the colon. Enteroendocrine cells of the GI tract constitute the largest endocrine organ in the body, collectively secreting more than 30 different hormones. Among the most physiologically important hormones released within the gastrointestinal tract are somatostatin, gastrin, ghrelin, secretin, glucagon-like peptide-1 (GLP-1), gastric inhibitory polypeptide (GIP), peptide YY (PYY), and cholecystokinin (CCK) [1,2,3,4].

The quantity of hormone secretion often reflects both the frequency and type of ingested meals. Each hormone has distinct sites of secretion within the GI tract. Some hormones are produced throughout the entire length of the intestine, while others are restricted to specific regions. For example, somatostatin is produced by endocrine cells in the pancreas. Ghrelin and gastrin are secreted by cells in the gastric mucosa. GLP-1 and PYY are predominantly found in the distal small intestine and colon, whereas GIP, secretin, and cholecystokinin are primarily localized in the proximal segments of the small intestine [3,5,6].

Gastrointestinal hormones play a key role in regulating numerous functions of the human body, influencing, among others, digestion, appetite, intestinal motility, and energy balance. Primarily, they control the sensation of hunger and satiety as well as food intake. Gastrin is released in response to food intake and plays a key role in suppressing hunger, stimulating gastric motility, and promoting gastric acid secretion. It also supports immune system function and stimulates the growth of the gastric mucosa. Under fasting conditions and in the absence of luminal nutrients, gastrin secretion is suppressed by somatostatin, which exerts its inhibitory effect through paracrine signaling. Somatostatin can also inhibit the secretion of other gastrointestinal hormones and, through its effects on the central nervous system, suppress the release of growth hormone. Furthermore, it promotes satiety mainly by inhibiting gastrointestinal peristalsis and delaying gastric emptying [4,5].

Ghrelin stimulates hunger and food intake, regulates the secretion of growth hormone, reduces energy expenditure, and promotes fat storage. In contrast, GLP-1 (glucagon-like peptide-1) is an incretin hormone that stimulates insulin release. It also enhances the sensation of satiety, inhibits food intake, and slows gastric emptying. GIP (glucose-dependent insulinotropic polypeptide) is another incretin hormone, which additionally reduces gastric acid secretion, decreases appetite, and suppresses food intake. Secretin primarily stimulates the pancreas to release bicarbonates and water, while inhibiting gastric acid secretion. Through this action, secretin creates an optimal environment for the activity of digestive enzymes. It also influences the central nervous system, contributing to the regulation of systemic water homeostasis. Peptide YY (PYY) suppresses appetite and food intake, slows intestinal peristalsis and gastric emptying, and decreases the secretion of pancreatic hormones. Cholecystokinin (CCK) stimulates intestinal motility and gallbladder contractions, promotes the release of digestive enzymes and insulin from the pancreas, enhances satiety, and reduces food intake [5,6,7].

Gastrointestinal hormones play a crucial role in the regulation of hunger and satiety by modulating the activity of the central nervous system (CNS). The hypothalamus and brainstem are key CNS structures involved in this regulation. They integrate afferent signals from peripheral organs, other brain regions, and the autonomic nervous system. These structures coordinate appropriate feedback mechanisms that enable the maintenance of energy homeostasis and an adaptive response to dynamic environmental changes [8,9]. The hypothalamus, located in the basal forebrain, is responsible for regulating metabolism, appetite, thermoregulation, and circadian rhythms. Hunger and satiety are primarily controlled by several hypothalamic nuclei, with the arcuate nucleus (ARC) playing a particularly central role. This nucleus contains two distinct populations of neurons. Neuropeptide Y (NPY) and agouti-related peptide (AgRP) neurons exert orexigenic (appetite-stimulating) effects, while pro-opiomelanocortin (POMC) and cocaine- and amphetamine-regulated transcript (CART) neurons mediate anorexigenic (satiety-inducing) responses. The activity of POMC/CART neurons is stimulated, among others, by GLP-1, whereas NPY/AgRP neurons may be activated by ghrelin [9]. Appetite-regulating signals reach the hypothalamus either directly, by crossing the blood–brain barrier, or indirectly, through binding to specific receptors on the vagus nerve. Upon reception of these peripheral and central inputs, hypothalamic neural circuits generate behavioral and physiological responses that modulate energy expenditure and nutrient intake, thereby maintaining systemic homeostasis [8].

Disruptions in the central nervous system pathways that regulate hunger and satiety contribute to a positive energy balance, increased adipose tissue accumulation, and overall body weight gain. However, it is important to emphasize that obesity is a multi-factorial disease. Its development is influenced by a complex interplay of genetic, environmental, behavioral, and psychological factors, which interact with one another [8]. Environmental and psychological factors may, among other mechanisms, influence the function of enteroendocrine cells located in the intestinal epithelium.

Several studies have shown that obesity is associated with altered secretion patterns of gastrointestinal hormones both in the fasting and postprandial state. Specifically, fasting levels of PYY and GLP-1 are often reduced in individuals with obesity compared with those with normal body weight, while their postprandial secretion is also blunted. In contrast, fasting ghrelin concentrations are typically lower in obesity, possibly due to the chronic positive energy balance, but the suppression of ghrelin after meal intake is delayed compared with lean individuals. These hormonal alterations may contribute to the development or maintenance of obesity or, alternatively, be a consequence of excess body weight [10].

These aspects are particularly relevant in the context of bariatric surgery, where changes in gastrointestinal hormone secretion are thought to play a key role in postoperative appetite regulation and metabolic improvements [10].

Nutrition is essential for human life and is closely linked to health. Every day, individuals make decisions regarding their dietary choices [11]. These choices are influenced not only by the perceived health benefits and potential risks of particular foods and beverages, but also by the pleasure associated with consumption and the level of satiety they induce [11]. Upon food intake, gastrointestinal activity is initiated. As digested food reaches the duodenum and small intestine, chemical substances and nutrients come into contact with enteroendocrine cells in the intestinal epithelium [12]. The primary dietary components that stimulate receptors on these cells are macronutrients: proteins, fats, and carbohydrates. This stimulatory effect becomes particularly evident after the digestive phase and is most pronounced during nutrient absorption [3]. In addition, the gut microbiota plays an important role in the regulation of food intake.

By metabolizing dietary compounds and producing specific metabolites, microorganisms colonizing the human gut play a key role in regulating intestinal barrier permeability and maintaining the integrity of the enteric nervous system. In addition, the bioactive substances they generate may act on multiple physiological systems, including the nervous, immune, and circulatory systems, as well as on intestinal enteroendocrine cells [2,13]. These interactions contribute to the regulation of energy and carbohydrate metabolism and influence the systemic inflammatory state. One of the most notable effects is the microbial fermentation of indigestible polysaccharides, which leads to the production of short-chain fatty acids (SCFAs). SCFAs have been shown to modulate appetite and satiety by influencing the secretion of gastrointestinal hormones. Moreover, the gut microbiota also produces secondary bile acids and indole derivatives, which further modulate the activity of enteroendocrine epithelial cells [2,14]. Among bacterial strains of particular interest is *Akkermansia muciniphila*. When administered exogenously, this species has demonstrated the capacity to reduce high-fat food intake, attenuate body weight and fat mass gain, enhance glucose tolerance, and improve intestinal barrier function. Recent studies also suggest that *A. muciniphila* may promote GLP-1 secretion, thereby supporting appetite regulation and glucose metabolism [15]. A deeper understanding of how gut microorganisms influence gastrointestinal hormone secretion may enable the development of novel, personalized nutritional strategies for the treatment of eating disorders and metabolic dysfunctions [16].

Physical activity is a multifactorial modulator of gastrointestinal function, gut microbiota composition, and the activity of intestinal enteroendocrine cells. In addition to a diet tailored to an individual’s energy needs—based on the resting metabolic rate—physical activity is essential for effective body weight reduction, particularly in reducing adipose tissue mass. Exercise has well-documented beneficial effects on the cardiovascular and respiratory systems, enhances muscular fitness, and improves overall quality of life. Furthermore, it exerts a positive influence on brain function, enhances sleep quality, supports emotional well-being, and alleviates symptoms of depression [17]. Notably, introducing regular physical activity may increase energy expenditure and counteract the adaptive reduction in metabolic rate that frequently occurs during weight loss. This effect is partly mediated by an increase in fat-free mass, especially skeletal muscle mass. Physical activity has also been shown to reduce appetite in individuals undergoing weight reduction and to decrease cravings for high-fat and high-sugar foods [18]. Regular physical activity has been linked to beneficial alterations in the secretion of hunger and satiety hormones, including a decrease in the levels of ghrelin and an increase in the concentrations of PYY, GLP-1, and leptin. These hormonal shifts are particularly significant and clinically relevant in individuals with obesity [19,20].

Obesity should not be viewed superficially as merely excess body fat or increased body weight. Rather, obesity is a chronic disease that involves complex dysfunctions of the human body, including psychological and emotional disturbances. Individuals with obesity frequently experience eating disorders, such as binge eating disorder and night-eating syndrome, as well as other mental health conditions, including depression and anxiety. It has been observed that obesity and its associated metabolic disturbances may contribute to the development of psychological disorders, and conversely, mental health conditions may exacerbate metabolic abnormalities. This bidirectional interplay between mental and metabolic health establishes a vicious cycle, wherein dysfunction in one domain exacerbates deterioration in the other. It is known that emotional dysregulation can influence gastrointestinal hormone secretion; however, the limited number of studies examining this link makes it difficult to specify the underlying mechanisms or draw definitive conclusions [21].

Although previous reviews have examined the role of gastrointestinal hormones in appetite regulation and obesity, most have focused on either a single hormone or specific intervention types (e.g., diet or bariatric surgery). However, a comprehensive overview that integrates the effects of multiple environmental factors—including diet composition, physical activity, and the gut microbiota—on gastrointestinal hormone secretion remains limited. In particular, the interaction between these modifiable factors and key hormones such as ghrelin, GLP-1, PYY, and CCK has not been sufficiently studied in the context of both normal-weight and obese individuals. Therefore, this narrative review aims to fill this gap by providing an up-to-date and integrated summary of how lifestyle and environmental influences modulate gastrointestinal hormone secretion, with special attention paid to implications for appetite regulation and obesity management.

Given the complexity of the gut endocrine system, which is influenced by numerous interrelated factors, this study focuses on a detailed analysis of the effects of dietary components, physical activity, and the gut microbiota on the release of gastrointestinal hormones.

## 2. Materials and Methods

A literature search was conducted in the PubMed database to identify relevant studies for this narrative review. The following search string was applied: (“VAT” OR “visceral adipose tissue” OR “hormones” OR “gastrointestinal hormones” OR “ghrelin” OR “GIP” OR “glucose-dependent insulinotropic peptide” OR “GLP-1” OR “glucagon-like peptide-1” OR “PYY” OR “peptide YY” OR “CCK” OR “cholecystokinin”) AND (“homeostasis” OR “functions” OR “metabolism” OR “physiology” OR “secretion” OR “morphology” OR “fat tissue” OR “nutrition” OR “microbiota” OR “physical activity”).

We aimed to limit the search to studies published within the last five years; however, in the absence of sufficient data, relevant older studies were also included. Out of approximately 3300 records initially identified, a total of 65 meta-analyses, reviews, and clinical studies were selected for inclusion in this article.

Among the wide range of gastrointestinal hormones, this review focuses on ghrelin, GLP-1, PYY, and CCK. These hormones were selected due to their well-established roles in appetite regulation and their frequent inclusion in clinical and experimental studies related to obesity, diet, and physical activity. Other hormones such as amylin, resistin, and adiponectin were not discussed in detail, as their involvement in gut–brain signaling is less extensively documented within the context of gastrointestinal secretion.

Only studies published in English with full-text availability were considered. Additional inclusion criteria comprised studies lasting longer than one day, involving both an experimental and a control group, and conducted exclusively in adult populations. In the absence of human data, relevant animal studies were considered.

**Figure 1:**
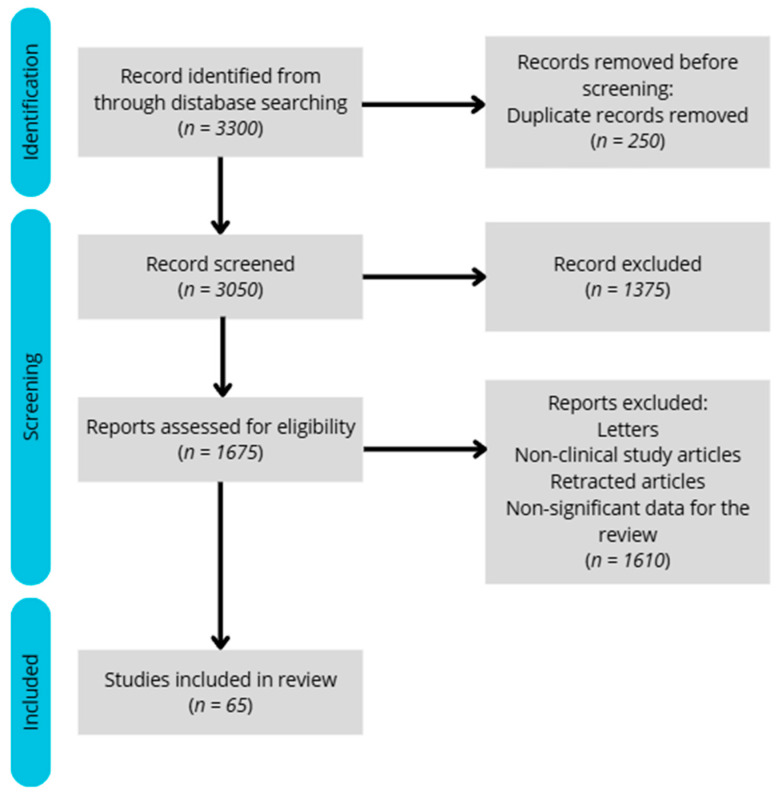
A flow diagram illustrating the process of article selection.

The included studies employed various methodologies, such as blood analysis, body composition assessments, and energy expenditure measurements. The exclusion criteria were as follows: lack of full-text access, imprecise results or study designs, studies shorter than 24 h, lack of a control group, studies involving children, or insufficient relevance to the topic. The flow diagram illustrating the study selection process for the review is presented below (Figure 1).

As the aim of this article was to provide a broad overview of factors affecting gastrointestinal hormone secretion, this review followed a narrative design and was not registered in the PROSPERO database.

## 3. Impact of Nutrition on Gastrointestinal Hormone Secretion

### 3.1. Ghrelin

Ghrelin is a 28-amino-acid peptide hormone and the only currently recognized orexigenic (appetite-stimulating) hormone. It is primarily secreted by X/A-like cells located in the gastric fundus, which represents its main site of production. These cells are the second most abundant type of endocrine cells in the stomach. In addition, ghrelin is secreted in smaller amounts in the pancreas, kidneys, intestines, pituitary gland, and heart. After being released, ghrelin undergoes enzymatic transformation, resulting in two forms: desacyl ghrelin and acyl ghrelin. Although desacyl ghrelin is more abundant in circulation, it is the acylated form that is biologically active as a hormone. Acyl ghrelin stimulates the release of growth hormone, plays a role in learning, memory, psychological stress, mood, and anxiety, and is involved in the sleep–wake cycle and aging. It also contributes to decreased insulin secretion and impaired glucose tolerance. However, ghrelin’s primary function is to stimulate appetite, and its secretion is closely related to food intake. Exogenous administration of ghrelin leads to weight gain, as it increases food consumption, promotes fat storage, and reduces energy expenditure. Ghrelin levels rise significantly before a meal and after fasting [22,23]. However, in individuals with obesity, ghrelin secretion is lower than in those with normal body weight. Moreover, fasting does not trigger an increase in ghrelin secretion, and ghrelin levels do not decrease after eating [24].

Ghrelin secretion is influenced by the composition of a meal. Previous studies have shown that proteins and carbohydrates have the greatest impact on reducing ghrelin levels, while fats appear to have a smaller effect. A study conducted by Tannous et al. [25] evaluated how acylated ghrelin concentrations change depending on the macronutrient composition of a meal. On different days, participants were given high-protein, high-fat, and high-carbohydrate meals. After each meal, a significant reduction in ghrelin levels was observed. However, the most pronounced immediate decrease occurred following the high-carbohydrate meal. In longer-term observations (180 min post-meal), the high-protein meal was most effective at maintaining low ghrelin levels. Based on these findings, the researchers concluded that increasing protein intake at the expense of carbohydrates and fats may beneficially affect the prolonged suppression of postprandial ghrelin secretion, thereby enhancing satiety [25].

These conclusions have also been confirmed in studies using animal models. Experimental studies in rats involving direct intragastrointestinal infusion of individual nutrients have demonstrated that glucose and amino acids elicit a more robust and rapid reduction in ghrelin levels compared with lipids. This attenuated ghrelin response to fats may underlie one of the physiological mechanisms contributing to increased energy intake and subsequent weight gain associated with high-fat diets [26].

The study by Hola et al. [24] demonstrated that a high-fat diet reduces ghrelin levels while simultaneously inducing resistance to its action. However, reverting to a standard dietary model normalizes ghrelin concentrations, improves ghrelin receptor sensitivity, and regulates metabolic parameters. The researchers emphasize that understanding the mechanisms by which dietary components influence ghrelin secretion provides clinicians with greater opportunities for treating obesity [24].

Moreover, not only the quantity of fat consumed but also its type appears to be important. A study by Prater et al. [27] investigated differences in ghrelin secretion following an 8-week dietary intervention depending on the type of oil consumed (cottonseed oil vs. olive oil). It was found that cottonseed oil suppressed postprandial ghrelin more strongly and for a longer duration compared with olive oil. This effect is attributed to differences in dominant fatty acids, indicating that polyunsaturated fatty acids (cottonseed oil) suppress ghrelin more effectively than monounsaturated fatty acids (olive oil) [27].

Additionally, studies have shown that after bariatric surgery (gastric bypass), ghrelin secretion becomes independent of the composition of the ingested food and does not differ compared with individuals who have not undergone the surgery [28].

Dietary fiber, particularly β-glucans found in oats, may also be beneficial in suppressing appetite and regulating glucose levels. A study by Gotteland et al. [29] demonstrated that β-glucans strongly inhibit appetite, in part, by suppressing ghrelin secretion. The researchers suggest that this component could be utilized in functional foods aimed at appetite and weight control [29].

In addition to meal composition, the regularity of food intake may influence ghrelin secretion. In a controlled six-week study conducted by Alhussain et al. [30], the impact of meal timing on ghrelin levels was evaluated. Participants adhered to a regular meal schedule during the initial two weeks, followed by a two-week washout phase, and subsequently engaged in irregular meal consumption for the final two weeks. The findings revealed that ghrelin suppression was more pronounced during periods of consistent meal timing. These results suggest that structured eating patterns may enhance the regulation of hunger and satiety, thereby contributing to improved weight management outcomes. However, it should be noted that the study sample was small (*n* = 9), indicating a need for further research with larger cohorts to confirm these findings [30].

In summary, carbohydrates exert the most substantial short-term effect on reducing ghrelin secretion, while proteins provide a more sustained long-term effect. β-glucans from oats also show promise in appetite suppression and may serve as effective components in functional food products. Nevertheless, some studies report conflicting results regarding the magnitude or timing of postprandial ghrelin suppression. These discrepancies may stem from variations in study populations (e.g., BMI, sex, or age), coexisting metabolic disorders, meal composition, and the timing of blood sampling, as well as differences in ghrelin assay sensitivity. Significant differences in results may also be caused by conducting tests on animals, which may not be reflected in humans. It is worth noting that the current research on the mechanisms regulating ghrelin secretion remains limited. Therefore, future studies stratified by nutritional status and metabolic health are warranted.

### 3.2. GIP

Glucose-dependent insulinotropic polypeptide (GIP) is a 42-amino acid peptide hormone. It is secreted by K cells located predominantly in the proximal duodenum and the initial part of the jejunum. GIP is classified as an incretin hormone, primarily responsible for stimulating insulin release from pancreatic β-cells. GIP receptors are also found in the central nervous system, particularly in the arcuate, paraventricular, and dorsomedial nuclei of the hypothalamus, indicating a role in appetite regulation. Through its central activity, GIP promotes satiety, contributing to energy homeostasis and body weight maintenance. GIP is predominantly secreted in response to food intake [31,32].

The first studies investigating the influence of dietary macronutrients on GIP secretion were conducted in the 1980s. In a study by Bailey et al. [33], which employed a murine model of obesity and hyperglycemia, researchers investigated the different effects of three distinct dietary interventions on GIP secretion. It was shown that a high-fat diet significantly increased GIP levels in both intestinal tissue and plasma compared with a standard diet. Additionally, there was an observed increase in the density of K cells. Although a carbohydrate-rich diet did not markedly alter GIP levels, it similarly led to an increased number of K cells. These results suggest that, among the tested interventions, a high-fat diet most strongly stimulates GIP secretion [33].

In a separate study, Yoder et al. [34] demonstrated that carbohydrates can also significantly enhance GIP secretion. Using a rat lymph fistula model, selective infusions of carbohydrates or proteins were administered. Carbohydrate administration resulted in a marked increase in GIP secretion, while protein infusion had no significant effect. The study applied five graded doses of proteins (0.08–1.29 g) and carbohydrates (0.07–1.1 g). The results showed a dose-dependent increase in GIP levels in response to carbohydrates, while none of the protein doses elicited significant changes. These findings underscore the insulinotropic potential of GIP in response to carbohydrate intake [34].

Further research has examined the effect of fasting on GIP levels. Deru et al. [35] found that GIP concentrations significantly decrease during fasting, whereas consumption of a high-fat meal strongly stimulates its secretion. However, no significant differences were observed between high-fat and high-carbohydrate meals in terms of GIP response. The authors noted that long-chain and monounsaturated fatty acids exert the strongest stimulatory effect on GIP secretion, while medium-chain and saturated fatty acids have only a minor influence. These outcomes may be partially attributed to the use of medium-chain saturated fats in the study. The authors suggested that high-fat meals may more effectively support appetite regulation than carbohydrate-rich meals but emphasized the need for further studies with more precisely controlled meal compositions [35].

GIP secretion may also be modulated by dietary fiber intake. In a study by Gotteland et al. [29], participants consumed a breakfast enriched with β-glucans derived from oats. It was shown that β-glucans enhance GIP secretion, potentially contributing to improved appetite control and weight regulation [29].

In conclusion, dietary protein appears to have little impact on GIP secretion by K cells. Carbohydrates and fats are significant stimulators of GIP release, with fats—particularly long-chain and monounsaturated fatty acids—exerting a stronger secretory effect. Moreover, dietary fiber, especially in the form of β-glucans, may also play an important role in modulating GIP secretion. Consideration of meal composition, with an emphasis on the type of macronutrients consumed, may be crucial for regulating GIP release, satiety, and glucose homeostasis. Further clinical studies on larger cohorts stratified by nutritional and metabolic profiles are required to fully elucidate the impact of individual macronutrients on GIP secretion. Although GIP is consistently stimulated by carbohydrate and lipid intake, its role in appetite regulation and energy balance remains unclear. Discrepancies in the literature—ranging from reports of exaggerated GIP responses in individuals with obesity to findings of no significant variation—may stem from differences in study design, insulin sensitivity, and participant characteristics.

### 3.3. GLP-1

Glucagon-like peptide-1 (GLP-1) is a peptide hormone composed of 29 amino acids. It is secreted by enteroendocrine L cells located in the mucosa of the ileum and colon. This hormone is released in two forms: GLP-1(7-37) and the amidated GLP-1(7-36). It has been observed that higher concentrations of the amidated form of GLP-1 are present in the circulation postprandially. Similar to glucose-dependent insulinotropic polypeptide (GIP), GLP-1 is an incretin hormone that stimulates pancreatic β-cells to release insulin. Additionally, it may enhance the survival of β-cells. This hormone also inhibits glucagon secretion, plays a significant role in delaying gastric emptying, and stimulates satiety at the hypothalamic level. It may also exert anti-inflammatory effects. GLP-1 secretion is particularly pronounced after meal ingestion. Its secretion follows a biphasic pattern, with the early phase occurring 10–15 min after a meal and the second, more prolonged phase occurring 30–60 min postprandially. The amount of hormone secreted can vary depending on the meal composition [31,36].

Protein intake has a significant effect on GLP-1 secretion regulation. This was demonstrated in a study by Hilkens et al. [37], in which participants replaced carbohydrates with increased amounts of protein from dairy products at breakfast. This modification resulted in increased GLP-1 concentrations, greater satiety, and improved glucose metabolism. The primary mechanisms by which GLP-1 influenced carbohydrate metabolism in this context were delayed gastric emptying and inhibition of glucagon secretion. The researchers also noted that not only protein in dairy but also other components, including calcium, positively affected GLP-1 levels. They emphasized that replacing carbohydrate-rich products with protein-rich foods, primarily dairy, may beneficially influence appetite control and, consequently, body weight regulation [37].

Crabtree et al. [38] also analyzed responses to high-protein versus normal-protein meals. The key focus was the analysis of GLP-1 levels relative to protein content in meals, as well as secretion patterns based on age, body weight, and sex. It was observed that high-protein meals more intensely stimulated GLP-1 release. No differences in GLP-1 concentration were noted based on body weight. Older individuals (mean age: 68 years) exhibited higher postprandial GLP-1 levels compared with younger adults (mean age: 31 years). GLP-1 secretion was stronger in women than in men. The researchers hypothesized that delayed gastric emptying could be a potential mechanism responsible for higher GLP-1 levels in these groups. This study provides a strong basis for further research into factors influencing hunger and satiety hormone secretion [38].

In a controlled clinical study, Neacsu et al. [39] investigated the differential postprandial effects of plant- versus animal-derived proteins on gastrointestinal hormone secretion. It was found that consumption of plant-based protein significantly increased serum GLP-1 levels compared with animal protein. These findings suggest that plant products can serve as valuable protein sources, supporting dietary recommendations to increase their daily intake. The authors proposed that replacing animal protein with plant protein—particularly from hemp—may beneficially regulate appetite via enhanced GLP-1 secretion. This mechanism may have potential applications in the dietary management of type 2 diabetes [39].

GLP-1 is effectively used in the treatment of patients with type 2 diabetes. One analysis examined serum GLP-1 concentrations following meals with varied macronutrient compositions, with a particular focus on carbohydrate-rich meals. Study participants consumed three different meals containing proteins, fats, and carbohydrates. A modest increase in GLP-1 concentration was observed, with the highest levels noted approximately 30 min after carbohydrate ingestion and 150 min after fat consumption. Subsequently, participants received 75 g of glucose or an equivalent portion of complex carbohydrates in the form of brown rice or barley groats. A significant GLP-1 increase was observed only after glucose administration. Monitoring for 24 h revealed that GLP-1 levels rose after all meals and remained elevated throughout the day, decreasing to baseline values only during nighttime. These results suggest that changes in GLP-1 secretion correspond to its physiological role as an incretin, and sustained elevated levels throughout the day may play a critical role in lipid anabolic metabolism [40].

To date, there are few studies investigating the impact of fats and high-fat diets on GLP-1 secretion. One such study by Parvaresh et al. [41] evaluated how GLP-1 secretion changes depending on meal composition. In this randomized crossover trial, GLP-1 secretion was significantly higher following high-fat and high-protein meals compared with high-carbohydrate meals. This effect was observed in both individuals with normal body weight and those with obesity. The researchers concluded that meals rich in protein or fat elicit more favorable hormonal responses related to hunger and satiety. This effect is observed in individuals with normal or excessive body weight as well as those with carbohydrate metabolism disorders [41].

In summary, the existing research results are varied. The secretion of GLP-1 following nutrient intake, especially protein, is documented, yet findings vary in magnitude and timing. Factors such as protein source, macronutrient combination, baseline metabolic state, and BMI may explain these differences. Additionally, differences in assay techniques and observation windows between studies further complicate direct comparisons. The most recent findings suggest that increasing dietary protein intake—particularly from dairy and plant sources—enhances GLP-1 secretion, which may improve appetite control, body weight management, and carbohydrate metabolism. Similarly, fat consumption may provide benefits in regulating hunger and satiety, especially in individuals with metabolic disorders. It is worth noting that earlier studies showed the most potent GLP-1 response after carbohydrate ingestion. Although the significant influence of dietary components on GLP-1 secretion is acknowledged, the number of studies elucidating the underlying mechanisms remains limited. Therefore, further research is needed to identify which nutrients have the greatest impact on GLP-1 secretion. Future studies should consider patients’ nutritional status, as body weight may affect postprandial GLP-1 release. Attention should also be given to meal size and form, as well as the rate of gastric emptying and intestinal transit. Such analyses will enable tailored nutritional therapy in the management of obesity and type 2 diabetes.

### 3.4. PYY

Peptide YY (PYY), similarly to GLP-1, is produced by L cells located within the mucosal lining of the ileum and colon. This hormone consists of 36 amino acids. The exact site of action of PYY remains unclear; however, electrophysiological studies involving administration into the arcuate nucleus suggest that PYY acts directly on hypothalamic NPY2 receptors. It has also been observed that PYY may exert effects via the brainstem and the vagus nerve. The central action of PYY strongly influences appetite suppression. It is hypothesized that PYY also affects gastric emptying, insulin secretion, and carbohydrate metabolism regulation. PYY secretion depends on both the quantity and composition of ingested meals. The satiety induced by this hormone may vary according to the amounts of protein, fat, and carbohydrates consumed [42,43,44].

Previous studies indicate that protein intake has the most potent effect on PYY secretion. Batterham et al. [42] demonstrated that high-protein meals produced the greatest reduction in hunger compared with high-carbohydrate and high-fat meals. This effect was accompanied by the highest increase in PYY concentrations relative to fasting levels, observed in both individuals with obesity and those with normal body weight. Notably, participants with normal body weight exhibited higher PYY concentrations than those with obesity [42].

The influence of protein on satiety was further confirmed in a study by Olivier et al. [45]. This study aimed to assess the effects of a high-protein diet versus a control diet on food intake regulation and the secretion of appetite-controlling hormones. The authors observed that, regardless of sex, consumption of a protein-rich diet led to increased PYY secretion. Consistent with prior findings, increased dietary protein intake was the main stimulus for PYY release. Additionally, a relationship was identified between energy balance and subjective satiety perception. These results underscore the significant role of protein in modulating PYY secretion and regulating food intake. They provide a foundation for further research on appetite control mechanisms and the potential application of high-protein diets in nutritional interventions [45].

Regarding the impact of diets with varying macronutrient compositions, fats have also been shown to significantly influence PYY secretion. Batterham et al. [42] evaluated the effects of high-protein, high-fat, and high-carbohydrate meals on PYY secretion. They found that meals enriched with fats stimulated PYY release to a greater extent than carbohydrate-rich meals. However, this effect was observed only in individuals with normal body weight. In the obese group, no significant changes in PYY concentrations were noted in response to fat intake. These findings suggest that the hormonal response to a high-fat diet is attenuated in obesity, potentially due to impaired appetite regulation and diminished anorexigenic effects of PYY [42]. Similar results were reported by Parvaresh et al. [41], who examined the effects of meals with different macronutrient compositions—high-protein, high-fat, and high-carbohydrate compositions—on PYY secretion in normal-weight and obese individuals. Carbohydrate-rich meals did not significantly influence PYY secretion in either group. In contrast, consumption of high-fat meals led to a marked increase in PYY levels regardless of nutritional status. Based on these findings, the authors suggest that replacing high-carbohydrate meals with fat- or protein-rich foods may be an effective nutritional strategy to enhance satiety and reduce spontaneous energy intake. This approach may be particularly relevant for obesity management [41].

In summary, although PYY is generally shown to increase postprandially, particularly after protein and fat intake, the strength and duration of the response are not uniform. Variability in study results may stem from differences in the amount and type of macronutrients, the energy content of meals, and participant characteristics such as age or weight status. This disparity may partially explain the altered satiety perception in obese patients, leading to excessive energy intake and further weight gain. Therefore, there is a need for in-depth, well-designed studies to elucidate the mechanisms regulating PYY secretion in response to different dietary components. Understanding these processes may be crucial for developing effective dietary strategies for individuals with overweight and obesity.

### 3.5. CCK

Cholecystokinin (CCK) is a 58-amino acid peptide hormone secreted by enteroendocrine I cells located in the duodenum and jejunum. It exerts its effects via two receptor subtypes found in the human body: CCKAR and CCKBR. CCK plays a pivotal role in promoting satiety and modulating digestive processes. Specifically, it regulates hepatic glucose production and stimulates gastric juice secretion, thereby influencing carbohydrate metabolism. It also induces gallbladder contraction [46]. The secretion of CCK is highly dependent on the composition of ingested meals. Fats are the most potent stimulators of CCK secretion. Polyunsaturated fatty acids (PUFAs) have been shown to significantly affect CCK secretion, as demonstrated in a study by Polley et al. [47] This randomized crossover trial aimed to assess the impact of polyunsaturated (PUFAs) versus monounsaturated fatty acids (MUFAs) on appetite and concentrations of hunger- and satiety-related hormones. Fifteen healthy male participants underwent two dietary interventions. Each intervention was preceded by a three-day run-in diet, followed by five days on a diet providing 50% of energy from fat, with either 25% from PUFAs or MUFAs. After each intervention, study visits were conducted involving test meals and an ad libitum meal. The results indicated that the PUFA-rich diet elicited higher postprandial CCK concentrations and greater subjective appetite suppression compared with the MUFA-rich diet, which did not produce similar effects [47]. Comparable outcomes were reported by Prater et al., where an eight-week dietary intervention involved daily consumption of cottonseed oil, rich in PUFAs, or olive oil, predominantly containing MUFAs. Both fasting and postprandial CCK levels were significantly higher in the cottonseed oil group than in the olive oil group. These findings suggest that cottonseed oil, due to its high PUFA content, may more effectively suppress appetite. This effect could be beneficial for body weight management, especially in individuals with metabolic disorders [27].

It is established that fats robustly stimulate CCK secretion, while proteins have a moderate effect. Carbohydrates appear to have the least effect on CCK secretion. This was confirmed by Seimon et al. [48], who administered duodenal infusions of mixtures differing in lipid-to-maltodextrin ratios. Ten healthy male participants underwent a randomized, double-blind crossover trial with three 90 min infusions: (1) 3 kcal/min lipids, (2) 2 kcal/min lipids + 1 kcal/min maltodextrin, and (3) 1 kcal/min lipids + 2 kcal/min maltodextrin. Hormonal responses, gastrointestinal motility, and appetite were assessed. The study revealed that decreasing lipid content in the infusions, accompanied by an increase in carbohydrates, led to a significant reduction in plasma CCK concentrations. This change was associated with increased food intake following the infusions. The authors concluded that lipids delivered to the gastrointestinal tract more strongly stimulate intestinal CCK secretion and more effectively reduce food intake compared with carbohydrates [48].

A similar study by Ryan et al. [49] involved twenty healthy, lean men participating in five randomized, double-blind sessions with 90 min duodenal infusions (3 kcal/min). The administered solutions varied as follows: (1) lipids only (3 kcal/min), (2) lipids and protein at a 2:1 ratio (2 kcal/min lipids + 1 kcal/min protein), (3) lipids and protein at a 1:2 ratio (1 kcal/min lipids + 2 kcal/min protein), (4) protein only (3 kcal/min), and (5) a saline solution (placebo). Hormonal responses, subjective appetite, and energy intake post-infusion were evaluated. The results demonstrated that all mixtures suppressed hunger; however, lipids most strongly stimulated CCK secretion. This suggests that proteins also influence CCK secretion and satiety, although to a lesser extent than lipids. The authors emphasized the need for further research into the mechanisms underlying the effects of individual macronutrients on CCK secretion [49].

**Figure 2:**
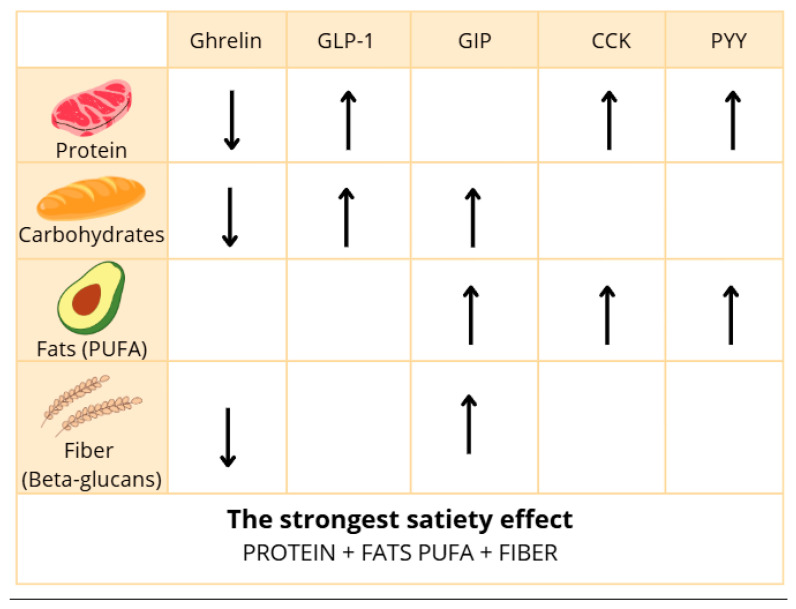
The impact of key nutrients on the secretion of gastrointestinal hormones. “↑”—increase secretion, “↓”—decrease secretion, and “ymbol of absence”—no consistent effect.

In summary, CCK is reliably released in response to fat and protein ingestion, yet some studies report inconsistent associations with appetite suppression. Discrepancies may result from variations in fat type (saturation and chain length), digestion rates, bile acid metabolism, and small sample sizes. Moreover, methodological heterogeneity limits direct comparison across trials. However, future studies focused on developing a precise methodology may allow us to assess whether CCK can be useful in controlling feelings of hunger and satiety. In addition, the results of future studies may help to control food intake and weight gain. The influence of dietary nutrients on the secretion of gastrointestinal hormones can vary. It depends on factors such as meal composition, frequency of consumption, meal size, and fiber content. The most important information is summarized in Figure 2 below.

## 4. Influence of Gut Microbiota on Gastrointestinal Hormone Secretion

The human gut microbiota constitutes a complex ecosystem of microorganisms, comprising at least 800 bacterial species, as well as fungi, archaea, protozoa, and viruses. Its composition dynamically changes, adapting to the organism’s conditions and needs. The microbiota of infants is relatively simple and primarily adapted for amino acid metabolism, whereas with age, changes occur that enable, among others, more efficient fat metabolism. Factors influencing its composition include age, dietary habits, lifestyle, and the presence of pathological conditions, including obesity. Aging is typically associated with a reduction in beneficial commensal bacteria and an increase in pathobionts and facultative anaerobes. These shifts contribute to disturbances in the Bacteroidetes-to-Firmicutes ratio, a dysbiosis that is particularly evident in individuals with obesity [50]. The gut microbiota metabolizes both dietary and endogenous substrates, generating bioactive compounds such as short-chain fatty acids (SCFAs) [51]. Research indicates that SCFAs produced by the gut microbiota can initiate a cascade of signals stimulating the secretion of gastrointestinal hormones. Acetate, one of the predominant SCFAs produced by gut microbial fermentation, exerts beneficial effects on energy metabolism by stimulating the release of GLP-1 and PYY. SCFAs act as ligands for the G protein-coupled receptor GPR41. Samuel et al. [52] demonstrated that mice lacking this receptor exhibited reduced PYY levels, suggesting that microorganisms, through SCFA production, impact satiety, food intake regulation, and overall energy metabolism [52]. The link between SCFAs and GLP-1 secretion was further confirmed by Tolhurst et al. [53], who, using a mouse model, showed that SCFAs act on FFAR2 receptors in L-cells, stimulating GLP-1 and PYY release. Mice deficient in these receptors displayed reduced GLP-1 expression and impaired glucose tolerance. The authors emphasize that this mechanism warrants detailed investigation in the context of metabolic disorders such as obesity and diabetes. They highlight FFAR2 receptors as potential pharmacological targets to stimulate the enteroendocrine system [53].

Although the gut microbiota has been shown to possess the capacity to synthesize tryptophan, its influence on gastrointestinal hormones remains ambiguous. Animal studies by Zhao et al. [54] showed that tryptophan supplementation increased ghrelin secretion, leading to heightened appetite and body weight gain [54]. Conversely, human studies demonstrated that intragastric or intraduodenal administration of tryptophan increased secretion of CCK, GLP-1, and PYY, resulting in appetite suppression and reduced energy intake [55,56,57]. Notably, while animal studies suggest tryptophan may stimulate ghrelin secretion and promote appetite, human studies consistently report enhanced release of satiety hormones such as CCK, GLP-1, and PYY, highlighting a potential species-specific divergence that warrants careful interpretation. This relationship requires further investigation, particularly regarding microbiota-derived tryptophan and its potential role in appetite regulation.

*Akkermansia muciniphila*, an anaerobic, Gram-negative gut bacterium first described in 2004, has gained increasing scientific attention in recent years. It primarily colonizes the mucus layer of the intestine and is capable of mucin degradation. Studies have shown that administration of live *A. muciniphila* cultures can stimulate endogenous production of antimicrobial peptides and bioactive lipids from the endocannabinoid family. These substances exhibit anti-inflammatory effects and influence GLP-1 and GLP-2 secretion [58,59].

Di et al. [60] demonstrated that the P9 protein produced by *A. muciniphila* binds to the ICAM-2 molecule, directly stimulating GLP-1 release [60]. Additionally, propionate (one of the SCFAs produced by this bacterium) also promotes GLP-1 and PYY secretion. Due to these properties, *A. muciniphila* plays a significant role in regulating appetite, food intake, and carbohydrate metabolism. Moreover, observations suggest that this bacterium may inhibit further weight gain in overweight individuals [58,61].

A substantial proportion of the current evidence on microbiota–hormone interactions, particularly regarding SCFAs and tryptophan, derives from animal studies. While these models provide mechanistic insights, significant physiological differences between species—such as microbiota composition, metabolism, and hormone signaling pathways—limit the direct translation of these findings to humans. Moreover, clinical studies in humans are still relatively few and often conducted in small cohorts, which challenges the generalizability of their outcomes. Therefore, care must be taken not to overinterpret these results until they are validated in larger, well-controlled human trials.

**Figure 3:**
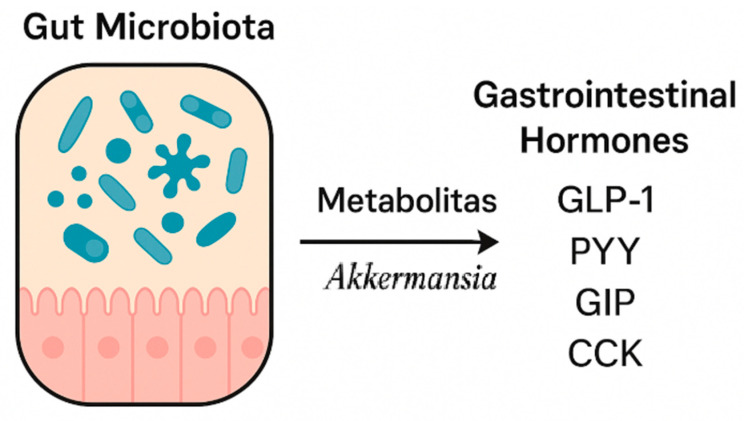
The impact of microbiota on the secretion of gastrointestinal hormones.

In summary (Figure 3), while SCFA-producing bacteria and certain probiotics are associated with improved hormone secretion profiles, the results are not universally consistent. Factors such as microbiota composition at baseline, dietary background, and inter-individual differences in fermentation capacity may influence outcomes. Furthermore, the majority of evidence stems from animal studies or small human cohorts, limiting generalizability. Despite accumulating evidence, further well-designed studies are needed, especially in populations with metabolic disorders. *Akkermansia muciniphila* may prove to be a particularly promising target for developing novel therapies supporting the treatment of obesity, diabetes, and other metabolic diseases. 

## 5. Impact of Physical Activity on Secretion of Gastrointestinal Hormones

Physical activity is widely recognized for its extensive health benefits. Although the underlying mechanisms through which it modulates physiological functions remain incompletely elucidated, one particularly interesting aspect is its impact on the secretion of gastrointestinal hormones involved in the regulation of appetite and food intake. A detailed meta-analysis of studies from the past 15 years was conducted by Mitoiu et al. [62], analyzing the effect of endurance exercise on ghrelin levels. The results of the reviewed studies were inconclusive. It was shown that long-term endurance training leads to an increase in ghrelin concentration, which constitutes a compensatory response to the reduction in body weight and fat mass. However, in overweight or obese individuals, short-term physical activity, regardless of type and intensity, did not cause significant changes in plasma ghrelin levels, both during and after exercise.

Regarding other appetite-regulating hormones, such as peptide YY (PYY) and glucagon-like peptide-1 (GLP-1), it was found that regular moderate-intensity physical activity contributes to an increase in GLP-1 concentration. This increase is associated with reduced feelings of hunger. At the same time, no significant changes in PYY levels were observed [62]. Additionally, Mitoiu et al. [62] reported that sprint interval training increases GLP-1 levels, leading to decreased hunger sensations without significant effects on the PYY concentration. However, the meta-analysis by Mitoiu et al. [62] included studies with varying protocols, exercise durations, and participant characteristics, which may explain the inconsistent results across hormones such as ghrelin, GLP-1, and PYY. Moreover, not all studies controlled for confounding variables such as baseline BMI, dietary intake, or sex, which limits comparability and may contribute to divergent hormonal responses. The secretion of these hormones may also depend on the exercise intensity. Intense resistance and aerobic exercise sessions lead to decreased ghrelin levels alongside increased PYY and GLP-1 concentrations. This effect contributes to appetite suppression and reduced energy intake [63]. This acute comparison of resistance vs. aerobic exercise was conducted in a small, physically inactive sample and found lower post-exercise GLP-1 and PYY levels despite greater ghrelin suppression, highlighting the need for larger and more diverse cohorts. Anderson et al. [64] investigated the influence of exercise intensity on ghrelin levels and hunger sensations in untrained individuals, with consideration of sex-based differences. Participants completed three cycling ergometer sessions of varying intensity, with appetite assessed using visual analog scales. The study demonstrated that high-intensity exercise elicited a more pronounced reduction in ghrelin concentrations and hunger ratings compared with moderate-intensity exercise, irrespective of sex. The authors emphasized that this suppressive effect occurs above the lactate threshold and highlighted the importance of considering hormones such as PYY and GLP-1 in appetite regulation [64]. Notably, this study’s findings on stronger ghrelin suppression with high-intensity exercise were limited to healthy, normal-weight adults, and sex differences emerged only for acyl-ghrelin in females, limiting generalizability to overweight populations.

**Figure 4:**
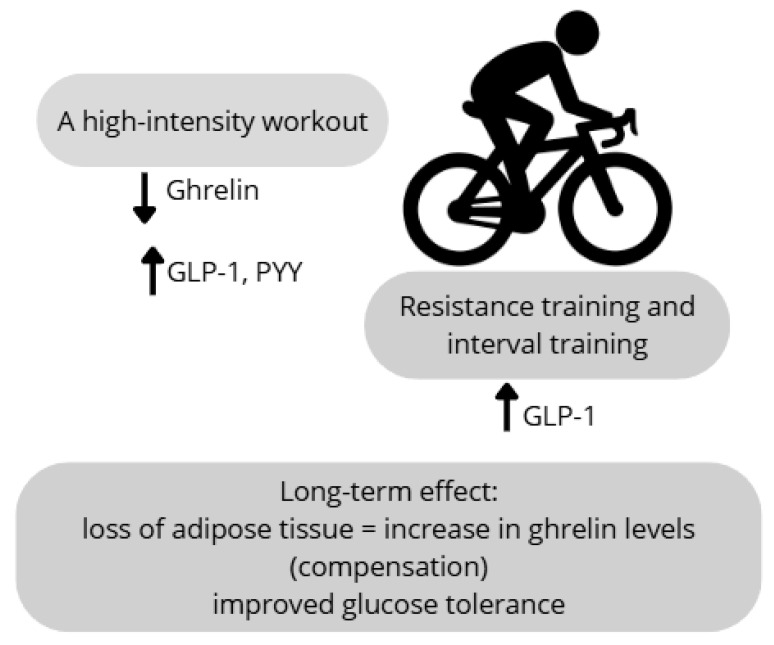
The impact of physical activity on the secretion of gastrointestinal hormones. “↑”—increased secretion; “↓”—decreased secretion.

Purcell et al. [65] analyzed the effect of weight loss on hunger and satiety hormone levels in overweight and obese subjects. Participants were divided into two groups: one underwent dietary intervention, and the other engaged in aerobic training. Both groups experienced significant weight loss, with no significant differences in weight reduction between groups. A compensatory increase in the ghrelin concentration was observed, though this change was not statistically significant. Similarly, non-significant decreases were noted for satiety hormones PYY and GLP-1. Based on these results, the authors concluded that the method of weight loss (diet versus physical activity) may not have a significant impact on hunger and satiety hormone levels. They also called for further, longer, and more detailed studies [65]. Although participants lost weight, the lack of significant differences in ghrelin, GLP-1, or PYY levels between diet and exercise groups may reflect the modest sample size, short follow-up, or similar energy deficits applied across groups. Although physical activity is recognized as a modulator of gastrointestinal hormone secretion, findings—particularly in overweight and obese populations—remain inconsistent and insufficiently characterized. The hormonal response to exercise is influenced by multiple factors, including the type, intensity, and duration of physical activity, as well as individual characteristics, such as body composition, nutritional status, and sex (Figure 4). Therefore, further research is warranted to determine the specific effects of various exercise modalities on the concentrations of appetite-regulating hormones. Future studies should incorporate study designs that account for these variables to optimize the therapeutic application of physical activity in the treatment of overweight and obesity.

To provide an integrated overview, we summarized the differential responses of the gastrointestinal hormones discussed in this review to various environmental and lifestyle factors, including macronutrient intake, microbial activity, and physical exercise (Table 1). This comparative perspective helps to highlight both shared and unique regulatory patterns that may have implications for appetite modulation and obesity management.

## 6. Conclusions

The accumulated data allow for several important conclusions regarding the influence of dietary components and physical activity on appetite regulation mediated by gastrointestinal hormones.

1. Carbohydrates exhibit an inhibitory effect on ghrelin secretion while simultaneously stimulating the release of satiety hormones such as GLP-1 and GIP, which may contribute to reduced feelings of hunger.
2. Proteins have a strong impact on appetite regulation by suppressing ghrelin secretion and promoting the release of CCK, PYY, and GLP-1, hormones responsible for satiety.
3. Fats also play a significant role in appetite control, mainly through the stimulation of CCK, PYY, GLP-1, and GIP secretion, resulting in appetite suppression.
4. Dietary fiber, although not directly digested, can influence appetite regulation, for instance, by inhibiting ghrelin secretion; however, further research on its effect on other gut hormones appears warranted.

These findings may be practically applied by healthcare professionals, including dietitians and physicians, in the development of individualized nutritional strategies aimed at appetite regulation. By incorporating macronutrients known to modulate gastrointestinal hormone secretion—such as proteins, dietary fiber, and unsaturated fats—into dietary plans, it is possible to support the management and prevention of obesity and metabolic disorders, including type 2 diabetes.

The growing understanding of the gut microbiota’s role in modulating gastrointestinal hormone secretion offers promising opportunities for the development of microbiota-targeted interventions—such as the use of prebiotics, probiotics, or dietary modifications—to support appetite regulation and metabolic health. Additionally, the recognition of physical activity as a modulator of hunger and satiety hormones highlights its potential as a complementary strategy in the prevention and treatment of overweight and obesity. However, given that the hormonal response to exercise varies depending on factors such as exercise type, intensity, duration, and individual characteristics (e.g., body mass, sex, and nutritional status), personalized approaches are essential to maximize its effectiveness.

It is important to highlight that the secretion patterns of gastrointestinal hormones are not uniform across populations with different body mass indices. Individuals with overweight or obesity often exhibit altered fasting and postprandial levels of hormones such as ghrelin, GLP-1, and PYY compared with normal-weight individuals. Therefore, body weight status should be considered a significant factor influencing hormone dynamics and the effectiveness of dietary or lifestyle interventions targeting appetite regulation.

Beyond their physiological significance, these findings may hold important translational potential. A more refined understanding of how dietary components, physical activity, and the gut microbiota influence gut hormone release may guide the development of personalized nutrition strategies for obesity prevention and treatment. Additionally, these conclusions can inform the design of future clinical trials targeting the gut–brain axis, using dietary, microbial, or behavioral interventions to modulate appetite and metabolic function in a way tailored to the patient’s needs. 

Future research should aim to better define how gastrointestinal hormone secretion is modulated in specific populations, particularly comparing individuals with obesity with those with normal body weight. Additionally, the synergistic effects of diet composition and physical activity on enteroendocrine responses remain underexplored and warrant further investigation in both short- and long-term interventions. There is also growing interest in identifying specific microbial strains or microbial-derived metabolites (e.g., SCFAs) that may serve as therapeutic targets for modulating hormone secretion. Interventional human studies will be essential to validate these mechanisms and support personalized strategies for appetite regulation and obesity prevention. 

## Figures and Tables

**Table 1:** Comparative overview of gastrointestinal hormone responses to environmental factors, including macronutrients, gut microbiota, and physical activity.

Hormone	Carbohydrates	Proteins	Fats	Microbiota	Physical Activity
Ghrelin	↓	↓	↓	↑	↓
GLP-1	↑	↑↑	↑	↑	↑
PYY	↑	↑↑	↑	↑	-/↑
GIP	↑↑	-	↑↑	↑	Data limited
CCK	-	↑	↑↑	↑	-

“↑”—increased secretion, “↑↑”—strong increase in secretion, “↓”—decreased secretion, and “-”—no consistent effect.

## Data Availability

No new data were created or analyzed in this study. Data sharing is not applicable to this article.

## Abbreviations

The following abbreviations were used in this manuscript:

AgRP	Agouti-related peptide
ARC	Arcuate nucleus
CART	Cocaine- and amphetamine-regulated transcript neurons
CCK	Cholecystokinin
CNS	Central nervous system
GI	Gastrointestinal
GIP	Gastric inhibitory polypeptide
GLP-1	Glucagon-like peptide-1
MUFA	Monounsaturated fatty acids
NPY	Neuropeptide Y
POMC	Pro-opiomelanocortin neurons
PUFAs	Polyunsaturated fatty acids
PYY	Peptide YY
SCFAs	Short-chain fatty acids
VAT	Visceral adipose tissue
